# The Impact of Maternal Age and Educational Level on Developmental Dysplasia of the Hip Diagnosis and Screening: A Descriptive Comparative Study

**DOI:** 10.1155/2023/6793645

**Published:** 2023-05-25

**Authors:** Moh'd S. Dawod, Mohammad N. Alswerki, Amr Z. Abuqudiri, Ashraf A. Albadaineh, Leena M. Mahmoud, Dania J. Altarawneh, Nour H. Rbeihat, Rogayah M. Alkhanazreh

**Affiliations:** ^1^Mutah University-Faculty of Medicine, Al-Karak, Jordan; ^2^Jordan University Hospital, Amman, Jordan; ^3^Ministry of Health, Al-Karak Hospital, Al-Karak, Jordan; ^4^Mutah University-Faculty of Medicine, Al-Karak, Jordan

## Abstract

**Background:**

Early and proper screening for developmental dysplasia of the hip (DDH) is very critical to prevent catastrophic complication on the developing hip joint. Many factors (either maternal or child-related) that hinder timely DDH screening have been previously investigated.

**Methods:**

A cross-sectional descriptive study design was adopted. 175 babies presented for DDH screening coming with their mothers were investigated. Maternal age, age group, and educational level were recorded. In addition, multiple child-related variables such as age of screening, gender, positive family history, preterm delivery, and mode of delivery were recorded as well. Analysis for association between delayed vs. early screening was made against the maternal and the child-related variables.

**Results:**

A total number of 175 children with their mothers were investigated. The mean maternal age was 27.9 years, about one third of the mothers had a graduate level of education (36.3%), while 41.1% had high school education, and 22.3% had middle school education. On the other hand, 40.0% of the investigated babies were first born and two thirds of our sample babies were females (66.9%). Of the included babies, 100 (57.1%) were screened at the appropriate 4-month age, while 75 (42.9%) missed the 4-month screening. Chi-square analysis showed that delayed DDH screening was associated with a lower maternal educational level (*P* ≤ 0.001), younger maternal age (*P* ≤ 0.001), and first born baby (*P* ≤ 0.001). Positive family history was protective against delayed DDH screening (*P* = 0.032).

**Conclusion:**

The lower maternal educational level, younger maternal age group, and first born babies are risk factors for delayed DDH screening.

## 1. Introduction

Developmental dysplasia of the hip (DDH) is considered one of the most common pediatric hip pathologies, affecting children very early in their musculoskeletal development [1, 2]. To avoid the devastating consequences of a missed DDH diagnosis, dysplastic hips should be diagnosed very early, before the hip joint has fully matured [3]. As a result, many countries have devised a screening protocol for the early detection and management of DDH [4, 5]. A portion of this screening is typically performed during the neonatal period using the Barlow and Ortolani hip screening exams [6]. A screening pelvic x-ray is routinely performed at the age of four months for screening, utilizing several radiological assessment angles [7–10].

Screening programs for DDH aim to facilitate early detection and treatment of the condition, and these programs vary significantly across the globe. In the United States, universal screening is recommended for all infants using physical examination techniques, including the Barlow and Ortolani maneuvers [11]. Conversely, in the United Kingdom, screening programs rely more heavily on targeting high-risk populations [12]. In contrast, Australia does not recommend routine screening using physical examination, instead focusing on high-risk populations, such as infants with a family history of DDH or those born in the breech position [13, 14]. Similarly, in Canada, universal screening is not recommended and screening is targeted towards high-risk populations using a combination of physical examination and imaging modalities [15]. These differences in screening programs can be attributed to various factors, such as cultural norms, healthcare infrastructure, and available resources. Ultimately, the effectiveness of screening programs for DDH may vary depending on the specific approach taken and the underlying characteristics of the population being screened.

Screening radiography is a highly successful method of avoiding missed or late dysplasia diagnoses. It is effective since it is simple and does not require any specific diagnostic tools or advanced diagnostic imaging modalities [16]. Furthermore, it has been shown to reduce child-related morbidity due to missed diagnoses [17]. A child who is diagnosed early can be treated with simple treatment modalities, whereas if the diagnosis is delayed, a more invasive treatment option, often surgical intervention, is required [18].

There are multiple barriers to DDH screening [14, 19, 20]. Some are related to the healthcare system's infrastructure, such as a lack of neonatal screening, a lack of an established organized screening program, a lack of trained pediatric or orthopedic specialists for diagnosis, and the inaccessibility of healthcare services due to insurance issues or children living in geographically remote areas [8, 21, 22]. But nonetheless, additional factors unrelated to the healthcare system, such as maternal sociodemographic factors, can be crucial.

Early detection and diagnosis of DDH are crucial for successful management. Fortunately, conservative management modalities such as the Pavlik Harness or abduction brace have a high success rate in managing DDH [23, 24]. However, timely diagnosis is paramount for effective treatment with these methods, as failure to detect DDH can render conservative management ineffective and necessitate surgical intervention [2]. Therefore, a proactive approach to screening and detection is essential to ensuring the optimal outcome for infants with DDH.

There are no previous studies that have investigated maternal influence on the screening for DDH; therefore, the aim of this study was to investigate some specific maternal factors that could lead to failure of screening and ultimately a delayed diagnosis of hip dysplasia.

## 2. Materials and Methods

A cross-sectional comparative study design was used in our study. A total of 175 mothers who presented to the pediatric orthopedic clinic from July 2022 to December 2022 for screening for DDH were included. Our inclusion criteria include all children who came with their mothers to the pediatric orthopedic clinic for screening during the prespecified study period. Exclusion criteria were as follows: children presented outside the specified study period; children not brought by their mothers; and children whose mothers were unable to provide consent for participation or could not provide complete medical histories of the babies.

From this cohort, 87 children were presented at the 4-month standard screening age and 88 children were presented lately after their children passed the 4-month screening age. All the presented children underwent screening using both physical examination assessment and screening pelvis x-ray to measure the acetabular index angle.

**Figure 1:**
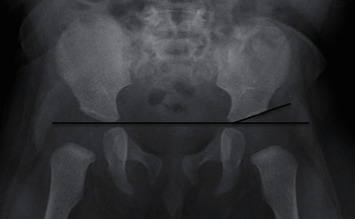
Normal anteroposterior radiograph of hips in 6-month-old boy shows that acetabular angles in the right and left hip (lines) are normal for age, measuring 22° and 24°, respectively (Starr et al. [25]).

**Figure 2:**
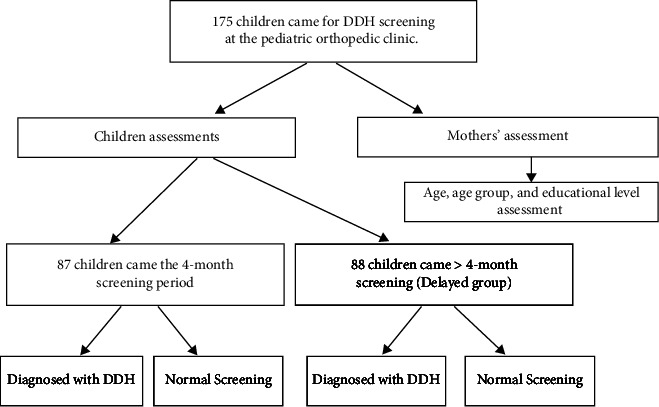
Summary of the research methodology.

An angle of inclination of more than 30 degrees was considered abnormal and diagnostic for DDH, and then appropriate orthopedic management was provided according to the patient's age. Angles below 30 degrees were considered normal, and routine follow-up was then established.  Figure 1 demonstrates the appropriate radiological technique to measure the acetabular index angles [25]. All patients were assessed and examined by an orthopedics consultant. The presenting mothers were assessed regarding the educational level, age group, and at the time of the clinic visit as well.  Figure 2 is an illustration of the study methodology.

Appropriate Institutional Review Board (IRB) for this study was obtained by the Mutah University Medical Research Office, IRB number (1072023). Appropriate informed consents were obtained from all participants of the study. The Code of Ethics of the World Medical Association (Declaration of Helsinki) was followed while conducting the study. Data regarding both mothers and patients were recorded and analyzed using the Statistical Package for Social Science (SPSS), version 23.

## 3. Results

The total number of included patients is 175 with their mothers. Data regarding maternal age and the educational level were obtained. Data regarding the DDH patients included age, gender, preterm delivery, neonatal intensive care unit admission, and family history of DDH.

**Figure 3:**
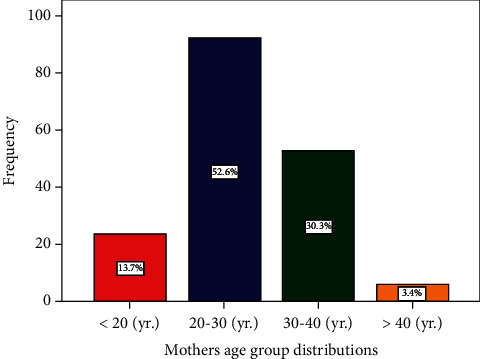
Distribution of maternal age groups.

**Figure 4:**
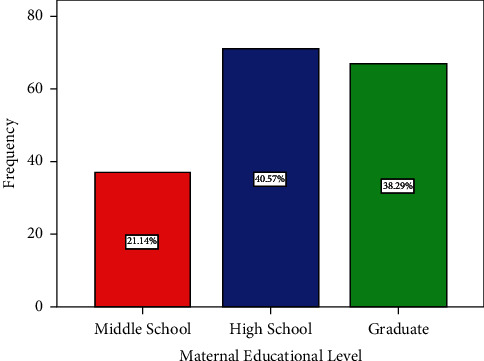
Distribution of maternal education level groups.

In terms of maternal data, the average age of moms was 27.9 years (SD 6.1). The mothers were divided into the four age categories listed as follows: the first group includes mothers under the age of 20 (24 mothers, 13.7%); the second group includes mothers between the ages of 20 and 30 (92 mothers, 52.6%); the third group includes mothers between the ages of 30 and 40 (53 mothers, 30.3%); and the last group includes mothers over 40 (6 mothers, 3.4%). As previously indicated, half of the mothers involved were between the ages of 20 and 30.  Figure 3 depicts a bar chart illustration of the maternal age distribution. As per the educational level of the included mothers, 39 mothers (22.3%) have middle school level education (school grades from 6th to 11th), 72 mothers have high school level education (41.1%), and nearly one-third of the mothers have graduate level education (36.3%).  Figure 4 depicts a bar chart illustration of the maternal educational level distribution.

Of the infants investigated, one-third (58 infants, 33.1%) were males and about two-thirds (117 infants, 66.9%) were females; fourteen (8%) of these infants were the result of twin conceptions. Seventy newborns were first-born (40.0%), whereas 44 were second-born (44.0%). Infants examined had a mean birth weight of 2.9 kilograms (SD 0.58). The delivery mode of the examined babies was nearly evenly distributed between normal spontaneous vaginal delivery (85 patients, 48.6%) and cesarean section (90 patients, 51.4%); 24 babies (13.7%) were delivered preterm (37 weeks of gestation), and 15 babies (8.6%) were admitted to neonatal critical care units.

Of the included babies, 100 (57.1%) were screened at the appropriate 4-month age, while 75 (42.9%) missed the 4-month screening age and presented for screening after this age. After screening all of the babies that came to the clinic, 87 (49.7%) were diagnosed with developmental dysplasia of the hip (DDH), whereas 88 (50.3%) had normal screening results. Twenty-four percent of all investee newborns have a family history of DDH, which means that at least one first-degree relative is affected. In terms of enteral feeding, approximately half of the newborns (90 or 51.4%) were breastfed, while the other half (85 or 48.6%) were fed formula.

An analysis was conducted using the chi-square test to test for potential associations between both the maternal educational level and the presentation for the 4-month standard national DDH screening. Our results showed a statistically significant association between the educational level of the mothers (*P* ≤ 0.001), the age of the mothers (*P* ≤ 0.001), and the baby being the first-born baby (*P* ≤ 0.001). This can be explained by the fact that the younger, less educated mothers, especially with their firstborn child, are most likely to be unaware of DDH diagnosis and the national standard 4-month screening. It is notable from the results that mothers with previous babies with DDH are more likely to bring their next baby to screening given their previous experience (*P* = 0.32). A comparison between the 4-month screened and delayed screening groups versus maternal factors is provided in Table 1, and a comparison versus child-related factors is provided in Table 2.

## 4. Discussion

Early detection and diagnosis via screening are crucial to preventing catastrophic complications in the developing hip join [26]. In this research, we examined the potential association among the maternal educational level, maternal age, and whether the baby was the first-born or not with the presentation of 4-month standard national DDH screening. The results revealed a statistically significant association among the maternal educational level, maternal age, and whether the baby was the first-born or not. Mothers who were younger, less educated, and had their first child were less likely to be aware of DDH diagnosis and the 4-month screening standard. However, mothers with higher education were more likely to bring their next baby for screening. Interestingly, over a third of the mothers in the study had graduate-level education, which is a promising sign of the importance of education and its potential impact on maternal and infant health outcomes. Maternal education is also linked to better health outcomes for the infant, as it may increase knowledge and practices related to infant health and wellness [27].

The current protocol for screening developmental dysplasia of the hip (DDH) in Jordan involves a two-stage process. The first stage involves neonatal physical examination screening immediately after birth, followed by screening radiography at four months of age. Should the infant present with abnormal physical examination results during the neonatal stage, a referral for ultrasonography is made, and the procedure is conducted by a consultant musculoskeletal radiologist. Subsequent management is implemented depending on the findings of the ultrasonography. Infants with normal examination results are re-evaluated at the four-month screening milestone. At the age of four months, screening is typically conducted at orthopedic outpatient clinics across hospitals within the healthcare sector. The diagnostic evaluation for DDH entails a comprehensive hip physical examination and a screening pelvis X-ray to measure the acetabular index. A normal acetabular index value is considered to be less than 30 degrees.

Our research study has shown an association between poor maternal education and delayed DDH screening, but the current screening approach in Jordan lacks emphasis on maternal education and counseling for DDH screening. As a result, this screening approach may contribute to delayed diagnosis and treatment of DDH, which could potentially affect our research study results. Our study findings suggest that there is a need for increased emphasis on maternal education and counseling to improve the screening and diagnosis of DDH in Jordan. By implementing such changes, we may improve the quality of care for infants at a risk for DDH and subsequently improve our research study results.

Since mothers are traditionally seen as the primary caregivers in our culture, we focused on investigating maternal-related variables that could potentially delay DDH screening. As a result of our study, we were capable of shedding light on maternal literacy and education level as a possible significant obstacle to delayed screening for DDH by the mothers.

Mulpuri et al., in their review of potential maternal risk factors for delayed DDH screening, reported that maternal age and parity were not associated with delayed presentation [28]. Sharpe et al. also showed that mother age, race, and number of prior pregnancies were not associated with delayed DDH screening in their investigation of variables associated with delayed screening [29]. We noticed a significant correlation between maternal age and delayed presentation in our maternal group. A possible reason is that, in our sample, the average maternal age for delayed presentation was younger than in Mulpuri's cohort (24 vs. 30 yrs.). So, the younger the mother, the less experience she has with the baby's health, which, when combined with the fact that a significant part of our cohort had only school-level education, led to a greater prevalence of delayed screening.

Our research revealed a statistically significant correlation between maternal education and the likelihood of a delayed presentation. As previously reported in the literature, the higher the maternal level of education, the greater the child health awareness, the better the child healthcare, and the greater the mother's access to healthcare systems [30, 31]. Therefore, these educationally disadvantaged mothers have poor healthcare knowledge regarding their babies, and this most likely led to delayed presentation. Notably, to the author's knowledge, no previous research has examined whether maternal education influences DDH screening.

In this research, our focus is on the educational level of mothers; thus, it is important to provide an overview of the general educational status in Jordan. According to the World Bank data, Jordan has achieved significant progress in enhancing its educational status, with a reported literacy rate of 96.1% and an average of 12.2 years of education as of 2021 [32]. While Jordan's educational status is higher than the regional average for the Middle East and North Africa, it falls slightly below the average for upper-middle-income countries [33]. Our research study on developmental dysplasia of the hip (DDH) screening in Jordan found that poor maternal education was strongly associated with delayed screening for DDH. This highlights the need to address gaps in maternal education and counseling for DDH.

Aside from maternal factors linked to delayed DDH screening, child-related factors have also been described in the literature. Lindberg et al. looked at the factors that can delay DDH screening and diagnosis. Late presentation was significantly associated with a negative family history, vertex pretension, and right-side dysplasia [34]. In their assessment of factors leading to delayed screening, Azzopardi et al. found that low birth weight, rural birth, and premature hospital release were associated with late screening and thus late diagnosis. [35]. In addition, female gender and normal delivery were risk factors for delayed screening, as reported by Sharpe et al. [29]. Habitual baby swaddling and cephalic presentation were related with an increased risk for late DDH screening in the study conducted by Mulpuri et al. [28]. In our study, we observed a substantial association between infant rank (if first born) and delayed DDH screening. According to the authors' best knowledge, this finding has not before been reported in the literature. Such findings can be rationalized by the likelihood that new mothers lack appropriate awareness of DDH screening. This probable explanation is supported by the fact that the frequency of screening delays decreases when a mother has more children, as illustrated by Table 2.

Despite some limitations, this study sheds light on the factors that contribute to delayed DDH screening. Although the sample size was small, it allowed for statistically significant associations to be detected between maternal education and delayed screening. While the study did not explore other potential influences such as cultural beliefs or access to healthcare, it is important to note that the study's main objective was to investigate the association between maternal education and delayed DDH screening. While the findings may not be generalizable to other regions or countries, they provide a useful reference for future research in similar populations. Overall, this study is a valuable contribution to the field, and further research is needed to fully understand the complexities of DDH screening.

## 5. Conclusion

Developmental hip dysplasia is a common pediatric orthopedic condition that affects children during their early hip joint development. Failure of early screening and, hence, early management will lead to early degenerative changes of the hip joint. Overcoming barriers to early DDH screening is an important topic to emphasize and analyze. The goal of our study was to highlight certain critical maternal characteristics that could have a detrimental impact on early screening, namely, maternal age and educational levels. Based on our findings, we may conclude that lower maternal educational levels and a younger age group were associated with delayed DDH screening. Mothers who care for their firstborn children are also at a higher risk.

## 6. Clinical Implications

In our study, the harsh negative effects of a poor maternal educational level on a critical child healthcare area were clearly emphasized and addressed. It is clear from our research how low an educational level was associated with delayed DDH screening. In addition, the same negative effects of a younger maternal group were strongly associated with delayed screening. Therefore, given that these previously mentioned variables led to jeopardized child healthcare in terms of proper DDH screening, the authors have some recommendations to help address this critical health issue.

1. Better DDH awareness, comprehensive health education, and screening counseling should be made available to mothers. This should be done early enough, especially in the third trimester of their pregnancy, as part of their prenatal care.
2. High-risk mothers, including those caring for their first babies and those with a lower educational profile, would benefit from close postpartum monitoring and scheduled appointments to orthopedic clinics in order to have better accessibility to screening.
3. Proper and timely screening for DDH should be approached as a multidisciplinary team. Involving primary healthcare providers and family physicians is advisable. Since these healthcare providers will be encountered by the mothers in the early healthcare of the babies (due to vaccination and early child healthcare establishment), involving such sectors will be a checkpoint to direct the mothers for early screening.

## 7. Unanswered Questions and Future Research

In this research, we shed light on some critical maternal characteristics that were associated with delayed DDH screening. In this study, however, we focused on just two of what we believed to be the most crucial potential risk factors for poor screening. Nonetheless, it is within the authors' scope to conduct additional follow-up research emphasizing other maternal characteristics such as locality (urban vs. rural), income level, and proximity to tertiary hospitals.

Furthermore, nonmaternal characteristics such as father profile, age, educational level, and child health awareness are worth investigating in future research. Another intriguing issue that may be addressed is the impact of having healthcare providers (nurses, doctors, and physical therapists) in the near family; would these affect early screening? It is also worth addressing that any family's misunderstandings about DDH diagnosis and treatment that may be impeding prompt screening. As mentioned, all this future follow-up research is within the scope of the authors' interests for near-future research.

## Figures and Tables

**Table 1:** Comparison between the 4-month screened and delayed screening groups versus maternal factors.

Maternal factor		4-month screened	Delayed screening	*P* value (differences among groups)
Maternal educational level	Middle school	12	25	*P* ≤ 0.001^*∗∗*^
	High school	39	32	
	Graduate	49	18	

Maternal age group	<20 (yr.)	3	21	*P* ≤ 0.001^*∗∗*^
	20–30 (yr.)	45	47	
	30–40 (yr.)	46	7	
	>40 (yr.)	6	0	
	Mean age (yr.)	24	31	0.04^*∗∗*^

**Table 2:** Comparison between the 4-month screened and delayed screening groups versus child-related factors.

Babies' factor	4-month screened	Delayed screening	*P* value (differences among groups)
Gender			
Male	31	27	0.48
Female	69	48	
Twins	8	6	0.99
Delivery mode			
NSVD	54	31	0.09
CS	46	44	
Preterm	13	11	0.75
NICU	9	6	0.81
FH	24	18	0.032^*∗∗*^
Breast feeding	53	37	0.63
Baby rank			
1	29	42	*P* ≤ 0.001^*∗∗*^
2	23	28	
3	17	2	
4	18	2	
5	7	0	
6	5	1	

NSVD: normal spontaneous vaginal delivery, CS: caesarian section, NICU: neonatal intensive care unit, and FH: family history.

## Data Availability

The data used to support the findings of this study are available from the corresponding author upon appropriate request.
